# A multilocus likelihood approach to joint modeling of linkage, parental diplotype and gene order in a full-sib family

**DOI:** 10.1186/1471-2156-5-20

**Published:** 2004-07-26

**Authors:** Qing Lu, Yuehua Cui, Rongling Wu

**Affiliations:** 1 Department of Statistics, University of Florida, Gainesville, Florida 32611 USA; 2 College of Life Sciences, Zhejiang Forestry University, Lin'an, Zhejiang 311300, People's Republic of China

## Abstract

**Background:**

Unlike a pedigree initiated with two inbred lines, a full-sib family derived from two outbred parents frequently has many different segregation types of markers whose linkage phases are not known prior to linkage analysis.

**Results:**

We formulate a general model of simultaneously estimating linkage, parental diplotype and gene order through multi-point analysis in a full-sib family. Our model is based on a multinomial mixture model taking into account different diplotypes and gene orders, weighted by their corresponding occurring probabilities. The EM algorithm is implemented to provide the maximum likelihood estimates of the linkage, parental diplotype and gene order over any type of markers.

**Conclusions:**

Through simulation studies, this model is found to be more computationally efficient compared with existing models for linkage mapping. We discuss the extension of the model and its implications for genome mapping in outcrossing species.

## Background

The construction of genetic linkage maps based on molecular markers has become a routine tool for comparative studies of genome structure and organization and the identification of loci affecting complex traits in different organisms [1]. Statistical methods for linkage analysis and map construction have been well developed in inbred line crosses [2] and implemented in the computer packages MAPMAKER [3], CRI-MAP [4], JOINMAP [5] and MULTIMAP [6]. Increasing efforts have been made to develop robust tools for analyzing marker data in outcrossing organisms [7-12], in which inbred lines are not available due to the heterozygous nature of these organisms and/or long-generation intervals.

Genetic analyses and statistical methods in outcrossing species are far more complicated than in species that can be selfed to produce inbred lines. There are two reasons for this. First, the number of marker alleles and the segregation pattern of marker genotypes may vary from locus to locus in outcrossing species, whereas an inbred line-initiated segregating population, such as an F_2 _or backcross, always has two alleles and a consistent segregation ratio across different markers. Second, linkage phases among different markers are not known *a priori *for outbred parents and, therefore, an algorithm should be developed to characterize a most likely linkage phase for linkage analysis.

To overcome these problems of linkage analysis in outcrossoing species, Grattapaglia and Sederoff [13] proposed a two-way pseudo-testcross mapping stratety in which one parent is heterozygous whereas the other is null for all markers. Using this strategy, two parent-specific linkage maps will be constructed. The limitation of the pseudo-testcross strategy is that it can only make use of a portion of molecular markers. Ritter et al. [7] and Ritter and Salamini [9] proposed statistical methods for estimating the recombination fractions between different segregation types of markers. Using both analytical and simulation approaches, Maliepaard et al. [10] discussed the power and precision of the estimation of the pairwise recombination fractions between markers. Wu et al. [11] formulated a multilocus likelihood approach to simultaneously estimate the linkage and linkage phases of the crossed parents over multiple markers. Ling [14] proposed a three-step analytical procedure for linkage analysis in out-crossing populations, which includes (1) determining the parental haplotypes for all of the markers in a linkage group, (2) estimating the recombination fractions, and (3) choosing a most likely marker order based on optimization analysis. This procedure was used to analyze segregating data in an outcrossing forest tree [15]. Currently, none of these models for linkage analysis in outcrossing species can provide a one-step analysis for the linkage, parental linkage phase and marker order from segregating marker data.

In this article, we construct a unifying likelihood analysis to simultaneously estimate linkage, linkage phases and gene order for a group of markers that display all possible segregation patterns in a full-sib family derived from two outbred parents (see Table 1 of Wu et al. [11]). Our idea here is to integrate all possible linkage phases between a pair of markers in the two parents, each specified by a phase probability, into the framework of a mixture statistical model. In characterizing a most likely linkage phase (or parental diplotype) based on the phase probabilities, the recombination fractions are also estimated using a likelihood approach. This integrative idea is extended to consider gene orders in a multilocus analysis, in which the probabilities of all possible gene orders are estimated and a most likely order is chosen, along with the estimation of the linkage and parental diplotype. We perform extensive simulation studies to investigate the robustness, power and precision of our statistical mapping method incorporating linkage, parental diplotype and gene orders. An example from the published literature is used to validate the application of our method to linkage analysis in outcrossing species.

**Table 1:** Estimation from two-point analysis of the recombination fraction ( ± SD) and the parental diplotype probability of parent P () and Q () for five markers in a full-sib family of *n *= 100

	Parental diplotype					*r *= 0.05			*r *= 0.20		
			
Marker	P^*a*^		×	Q^*a*^							
	|	|		|	|						
	*a*	*b*		*c*	*d*						
	|	|		|	|	0.530 ± 0.0183			0.2097 ± 0.0328		
	*a*	*b*		*a*	*b*		0.9960	0.9972		0.9882	0.9878
	|	|		|	|	0.0464 ± 0.0303			0.2103 ± 0.0848		
	*a*	*o*	×	*o*	*a*		1 (0^*b*^)	0(1^*b*^)		1 (0^*b*^)	0(1^*b*^)
	|	|		|	|	0.0463 ± 0.0371			0.1952 ± 0.0777		
	*a*	*b*		*b*	*b*		1	1/0^*c*^		1	1/0^*c*^
	|	|		|	|	0.0503 ± 0.0231			0.2002 ± 0.0414		
	*a*	*b*		*c*	*d*		1	1/0^*c*^		1	1/0^*c*^

^*a*^Shown is the parental diplotype of each parent for the five markers hypothesized, where the vertical lines denote the two homologous chromosomes. ^*b*^The values in the parentheses present a second possible solution. For any two symmetrical markers (2 and 3),  = 1,  = 0 and  = 0,  = 1 give an identical likelihood ratio test statistic (Wu et al. 2002a). Thus, when the two parents have different diplotypes for symmetrical markers, their parental diplotypes cannot be correctly determined from two-point analysis. ^*c*^The parental diplotype of parent *P*_2 _cannot be estimated in these two cases because marker 4 is homozygous in this parent. The MLE of *r *is given between two markers under comparison, whereas the MLEs of *p *and *q *given at the second marker.

### Two-locus analysis

#### A general framework

Suppose there is a full-sib family of size *n *derived from two outcrossed parents P and Q. Two sets of chromosomes are coded as **1 **and **2 **for parent P and **3 **and **4 **for parent Q. Consider two marker loci  and , whose genotypes are denoted as 12/12 and 34/34 for parent P and Q, respectively, where we use / to separate the two markers. When the two parents are crossed, we have four different progeny genotypes at each marker, i.e., 13, 14, 23 and 24, in the full-sib family. Let *r *be the recombination fraction between the two markers.

In general, the genotypes of the two markers for the two parents can be observed in a molecular experiment, but the allelic arrangement of the two markers in the two homologous chromosomes of each parent (i.e., linkage phase) is not known. In the current genetic literatuire, a linear arrangement of nonalleles from different markers on the same chromosomal region is called the *haplotype*. The observable two-marker genotype of parent P is 12/12, but it may be derived from one of two possible combinations of maternally- and paternally-derived haplotypes, i.e., [11] [22] or [12] [21], where we use [] to define a haplotype. The combination of two haplotypes is called the *diplotype*. Diplotype [11] [22] (denoted by 1) is generated due to the combination of two-marker haplotypes [11] and [22], whereas diplotype [12] [21] (denoted by ) is generated due to the combination of two-marker haplotypes [12] and [21]. If the probability of forming diplotype [11] [22] is *p*, then the probability of forming diplotype [12] [21] is 1 - *p*. The genotype of parent Q and its possible diplotypes [33] [44] and [34] [43] can be defined analogously; the formation probabilities of the two diplotypes are *q *and 1 - *q*, respectively.

The cross of the two parents should be one and only one of four possible parental diplotype combinations, i.e., [11] [22] × [33] [44]), [11] [22] × [34] [43], [12] [21] × [33] [44] and [12] [21] × [34] [43], expressed as 11, 1, 1 and , with a probability of *pq*, *p*(1 - *q*), (1 - *p*)*q *and (1 - *p*) (1 - *q*), respectively. The estimation of the recombination fraction in the full-sib family should be based on a correct diplotype combination [10]. The four combinations each will generate 16 two-marker progeny genotypes, whose frequencies are expressed, in a 4 × 4 matrix, as



for [11] [22] × [33] [44],



for [11] [22] × [34] [43],



for [12] [21] × [33] [44] and



for [12] [21] × [34] [43]. Note that these matrices are expressed in terms of the combinations of the progeny genotypes for two markers  and , respectively.

Let **n **= (*n*_*j*1*j*2_)_4 × 4 _denote the matrix for the observations of progeny where *j*_1_,*j*_2 _= 1 for 13, 2 for 14, 3 for 23, or 4 for 34 for the progeny genotypes at these two markers. Under each parental diplotype combination, *n*_*j*1*j*2 _follows a multinomial distribution. The likelihoods for the four diplotype combinations are expressed as



where *N*_1 _= *n*_11 _+ *n*_22 _+ *n*_33 _+ *n*_44_, *N*_2 _= *n*_14 _+ *n*_23 _+ *n*_32 _+ *n*_41_, *N*_3 _= *n*_12 _+ *n*_21 _+ *n*_34 _+ *n*_43_, and *N*_4 _= *n*_13 _+ *n*_31 _+ *n*_24 _+ *n*_42_. It can be seen that the maximum likeihood estimate (MLE) of r () under the first diplotype combination is equal to one minus  under the fourth combination, and the same relation holds between the second and third diplotype combinations. Although there are identical plug-in likelihood values between the first and fourth combinatins as well as between the second and third combinations, one can still choose an appropriate  from these two pairs because one of them leads to  greater than 0.5. Traditional approaches for estimating the linkage and parental diplotypes are to estimate the recombination fractions and likelihood values under each of the four combinations and choose one legitimate estimate of *r *with a higher likelihood.

In this study, we incorporate the four parental diplotype combinations into the observed data likelihood, expressed as



where **Θ **= (*r*, *p*, *q*) is an unknown parameter vector, which can be estimated by differentiating the likelihood with respect to each unknown parameter, setting the derivatives equal to zero and solving the likelihood equations. This estimation procedure can be implemented with the EM algorithm [2,11,16]. Let **H **be a mixture matrix of the genotype frequencies under the four parental diplotype combinations weighted by the occurring probabilities of the diplotype combinations, expressed as



where



Similar to the expression of the genotype frequencies as a mixture of the four diplotype combinations, the expected number of recombination events contained within each two-marker progeny genotype is the mixture of the four different diplotype combinations, i.e.,



where the expected number of recombination events for each combination are expressed as



Define



The general procedure underlying the {*τ *+ 1}th EM step is given as follows:

**E Step: **At step *τ*, using the matrix **H **based on the current estimate *r*^{*τ*}^, calculate the expected number of recombination events between two markers for each progeny genotype and ,







where *d*_*j*1*j*2_, *h*_*j*1*j*2_, *p*_*j*1*j*2 _and *q*_*j*1*j*2 _are the (j_1_*j*_2_)th element of matrix **D**, **H**, **P **and **Q**, respectively.

**M Step: **Calculate *r*^{*τ*+1} ^using the equation,



The E step and M step among Eqs. (4) – (7) are repeated until *r *converges to a value with satisfied precision. The converged values are regarded as the MLEs of **Θ**.

#### Model for partially informative markers

Unlike an inbred line cross, a full-sib family may have many different marker segregation types. We symbolize observed marker alleles in a full-sib family by *A*_1_, *A*_2_, *A*_3 _and *A*_4_, which are codominant to each other but dominant to the null allele, symbolized by *O*. Wu et al. [11] listed a total of 28 segregation types, which are classified into 7 groups based on the amount of information for linkage analysis:

A. Loci that are heterozygous in both parents and segregate in a 1:1:1:1 ratio, involving either four alleles *A*_1_*A*_2 _× *A*_3_*A*_4_, three non-null alleles *A*_1_*A*_2 _× *A*_1_*A*_3_, three non-null alleles and a null allele *A*_1_*A*_2 _× *A*_3_*O*, or two null alleles and two non-null alleles *A*_1_*O *× *A*_2_*O*;

B. Loci that are heterozygous in both parents and segregate in a 1:2:1 ratio, which include three groups:

B_1_. One parent has two different dominant alleles and the other has one dominant allele and one null allele, e.g., *A*_1_*A*_2 _× *A*_1_*O*;

B_2_. The reciprocal of B_1_;

B_3_. Both parents have the same genotype of two codominant alleles, i.e., *A*_1_*A*_2 _× *A*_1_*A*_2_;

C. Loci that are heterozygous in both parents and segregate in a 3:1 ratio, i.e., *A*_1_*O *× *A*_1_*O*;

D. Loci that are in the testcross configuration between the parents and segregate in a 1:1 ratio, which include two groups:

D_1_. Heterozygous in one parent and homozygous in the other, including three alleles *A*_1_*A*_2 _× *A*_3_*A*_3_, two alleles *A*_1_*A*_2 _× *A*_1_*A*_1_, *A*_1_*A*_2 _× *OO *and *A*_2_*O *× *A*_1_*A*_1_, and one allele (with three null alleles) *A*_1_*O *× *OO*;

D_2_. The reciprocals of D_1_.

The marker group A is regarded as containing *fully informative *markers because of the complete distinction of the four progeny genotypes. The other six groups all contain the *partially informative *markers since some progeny genotype cannot be phenotypically separated from other genotypes. This incomplete distinction leads to the segregation ratios 1:2:1 (B), 3:1 (C) and 1:1 (D). Note that marker group D can be viewed as fully informative if we are only interested in the heterozygous parent.

In the preceding section, we defined a (4 × 4)-matrix **H **for joint *genotype *frequencies between two fully informative markers. But for partially informative markers, only the joint *phenotypes *can be observed and, thus, the joint genotype frequencies, as shown in **H**, will be collapsed according to the same phenotype. Wu et al. [11] designed specific incidence matrices (**I**) relating the genotype frequencies to the phenotype frequencies for different types of markers. Here, we use the notation  for a (*b*_1 _× *b*_2_) matrix of the phenotype frequencies between two partially informative markers, where *b*_1 _and *b*_2 _are the numbers of distinguishable phenotypes for markers  and , respectively. Correspondingly, we have . The EM algorithm can then be developed to estimate the recombination fraction between any two partial informative markers.

**E Step: **At step *τ*, based on the matrix (**DH**)' derived from the current estimate *r*^{*τ*}^, calculate the expected number of recombination events between the two markers for a given progeny genotype and :







where , ,  and  is the (*j*_1_*j*_2_)th element of matrices (**DH**)', **H**', **P**' and **Q**', respectively.

**M Step**: Calculate *r*^{*τ*+1} ^using the equation,



The E and M steps between Eqs. (8) – (11) are repeated until the estimate converges to a stable value.

### Three-locus analysis

#### A general framework

Consider three markers in a linkage group that have three possible orders ,  and . Let *o*_1_, *o*_2 _and *o*_3 _be the corresponding probabilities of occurrence of these orders in the parental genome. Without loss of generality, for a given order, the allelic arrangement of the first marker between the two homologous chromosomes can be fixed for a parent. Thus, the change of the allelic arrangements at the other two markers will lead to 2 × 2 = 4 parental diplotypes. The three-marker genotype of parent P (12/12/12) may have four possible diplotypes, [111] [222], [112] [221], [121] [212] and [122] [211]. Relative to the fixed allelic arrangement 1|2| of the first marker on the two homologous chromosomes **1 **and **2**, the probabilities of allelic arragments 1|2| and 2|1| are denoted as *p*_1 _and 1 - *p*_1 _for the second marker and as *p*_2 _and 1 - *p*_2 _for the third marker, respectively. Assuming that allelic arrangements are independent between the second and third marker, the probabilities of these four three-marker diplotypes can be described by *p*_1_*p*_2_, *p*_1_(1 - *p*_2_), (1 - *p*_1_)*p*_2 _and (1 - *p*_1_) (1 - *p*_2_), respectively. The four diplotypes of parent Q can also be constructed, whose probabilities are defined as *q*_1_*q*_2_, *q*_1_(1 - *q*_2_), (1 - *q*_1_)*q*_2 _and (1 - *q*_1_) (1 - *q*_2_) respectively. Thus, there are 4 × 4 = 16 possible diplotype combinations (whose probabilities are the product of the corresponding diplotype probabilities) when parents P and Q are crossed.

Let *r*_12 _denote the recombination fraction between markers  and , with *r*_23 _and *r*_13_defined similarly. These recombination fractions are associated with the probabilities with which a crossover occurs between markers  and  and between markers  and . The event that a crossover or no crossover occurs in each interval is denoted by *D*_11 _and *D*_00_, respectively, whereas the events that a crossover occurs only in the first interval or in the second interval is denoted by *D*_10 _and *D*_01_, respectively. The probabilities of these events are denoted by *d*_00_, *d*_01_, *d*_10_and *d*_11_, respectively, whose sum equals 1. According to the definition of recombination fraction as the probability of a crossover between a pair of loci, we have *r*_12 _= *d*_10 _+ *d*_11_, *r*_23 _= *d*_01 _+ *d*_11 _and *r*_13 _= *d*_01 _+ *d*_10_. These relationships have been used by Haldane [17] to derive the map function that converts the recombination fraction to the corresponsding genetic distance.

For a three-point analysis, there are a total of 16 (16 × 4)-matrices for genotype frequencies under a given marker order (), each corresponding to a diplotype combination, denoted by , where  for 1|2| or 2 for 2|1| denote the two alternative allelic arrangements of the second and third marker, respectively, for parent P, and  for 1|2| or 2 for 2|1| denote the two alternative allelic arrangements of the second and third marker, respectively, for parent Q. According to Ridout et al. [18] and Wu et al. [11], elements in  are expressed in terms of *d*_00_, *d*_01_, *d*_10 _and *d*_11_.

Similarly, there are 16 (16 × 4)-matrices for the expected numbers of crossover that have occurred for *D*_00_, *D*_01_, *D*_10 _and *D*_11 _for a given marker order, denoted by , ,  and  respectively. In their Table 2, Wu et al. [11] gave the three-locus genotype frequencies and the number of crossovers on different marker intervals under marker order .

**Table 2:** Estimation from three-point analysis of the recombination fraction ( ± SD) and the parental diplotype probabilities of parent P () and Q () for five markers in a full-sib family of *n *= 100

	Parental diplotype												
							
Marker	P		×	Q		Case 1	Case 2			Case 1	Case 2		
Recombination fraction = 0.05													
	|	|		|	|								
	*a*	*b*		*c*	*d*								
	|	|		|	|	0.0511 ± 0.0175							
	*a*	*b*		*a*	*b*		0.1008 ± 0.0298	0.9978	0.9986				
	|	|		|	|	0.0578 ± 0.0269				0.0557 ± 0.0312			
	*a*	*o*	×	*o*	*a*			0.9977	0		0.0988 ± 0.0277	1	0
	|	|		|	|	0.0512 ± 0.0307				0.0476 ± 0.0280		1	1/0
	*a*	*b*		*b*	*b*		0.0932 ± 0.0301	1	1/0			1	1/0
	|	|		|	|	0.0514 ± 0.0229							
	*a*	*b*		*c*	*d*			1	1				
	|	|		|	|								

Recombination fraction = 0.20													
	|	|		|	|								
	*a*	*b*		*c*	*d*								
	|	|		|	|	0.2026 ± 0.0348							
	*a*	*b*		*a*	*b*		0.3282 ± 0.0482	0.9918	0.9916				
	|	|		|	|	0.2240 ± 0.0758				0.2408 ± 0.0939			
	*a*	*o*	×	*o*	*a*			0.9944	0		0.3241 ± 0.0488	1	0
	|	|		|	|	0.1927 ± 0.0613				0.1824 ± 0.0614			
	*a*	*b*		*b*	*b*		0.3161 ± 0.0502	1	1/0			1	1/0
	|	|		|	|	0.2017 ± 0.0393							
	*a*	*b*		*c*	*d*			1	1				
	|	|		|	|								

Case 1 denotes the recombination fraction between two adjacent markers, whereas case 2 denotes the recombination fraction between the two markers separated by a third marker. See Table 1 for other explanations.

The joint genotype frequencies of the three markers can be viewed as a mixture of 16 diplotype combinations and three orders, weighted by their occurring probabilities, and is expressed as



Similarly, the expected number of recombination events contained within a progeny genotype is the mixture of the different diplotype and order combinations, expressed as:









Also define



The occurring probabilities of the three marker orders are the mixture of all diplotype combinations, expressed, in matrix notation, as



We implement the EM algorithm to estimate the MLEs of the recombination fractions between the three markers. The general equations formulating the iteration of the {*τ *+ 1}th EM step are given as follows:

**E Step: **As step *τ*, calculate the expected number of recombination events associated with *D*_00_(*α*), *D*_01 _(*β*), *D*_10_(*γ*), *D*_11_(*δ*) for the (*j*_1_*j*_2_*j*_3_)th progeny genotype (where *j*_1_, *j*_2 _and *j*_3 _denote the progeny genotypes of the three individual markers, respectively):









Calculate , , ,  and , (*k *= 1,2,3) using











where *n*_*j*1*j*2*j*3 _denote the number of progeny with a particular three-marker genotype, h_*j*1*j*2*j*3_, , , , , *p*_1(*j*1*j*2*j*3)_, *p*_2(*j*1*j*2*j*3)_, *q*_1(*j*1*j*2*j*3) _and *q*_2(*j*1*j*2*j*3) _are the (*j*_1_*j*_2_*j*_3_)th element of matrices **H**, **D**_00_, **D**_01_, **D**_10_, **D**_11_, **P**_1_, **P**_2_, **Q**_1 _and **Q**_2_, respectively.

**M Step: **Calculate , ,  and  using the equations,









The E and M steps are repeated among Eqs. (19) – (32) until *d*_00_, *d*_01_, *d*_10 _and *d*_11 _converge to values with satisfied precision. From the MLEs of the *g's*, the MLEs of recombination fractions *r*_12_, *r*_13 _and *r*_23 _can be obtained according to the invariance property of the MLEs.

#### Model for partial informative markers

Consider three partially informative markers with the numbers of distinguishable pheno-types denoted by *b*_1_, *b*_2 _and *b*_3_, respectively. Define  is a (*b*_1_*b*_2 _× *b*_3_) matrix of genotype frequencies for three partially informative markers. Similarly, we define , and .

Using the procedure described in Section (2.2), we implement the EM algorithm to estimate the MLEs of the recombination fractions among the three partially informative markers.

#### m-point analysis

Three-point analysis considering the dependence of recombination events among different marker intervals can be extended to perform the linkage analysis of an arbitrary number of markers. Suppose there are *m *ordered markers on a linkage group. The joint genotype probabilities of the *m *markers form a (4^*m*-1 ^× 4)-dimensional matrix. There are 2^*m*-1 ^× 2^*m*-1 ^such probability matrices each corresponding to a different parental diplotype combination. The reasonable estimates of the recombination fractions rely upon the characterization of a most likely parental diplotype combination based on the multilocus likelihood values calculated.

The *m*-marker joint genotype probabilities can be expressed as a function of the probability of whether or not there is a crossover occurring between two adjacent markers,  where *l*_1_, *l*_2_, ..., *l*_*m*-1 _are the indicator variables denoting the crossover event between markers  and , markers  and , ..., and markers  and , respectively. An indicator is defined as 1 if there is a crossover and 0 otherwise. Because each indicator can be taken as one or zero, there are a total of 2^*m*-1 ^D's.

The occurring probability of interval-specific crossover  can be estimated using the EM algorithm. In the E step, the expected number of interval specific crossovers is calculated (see Eqs. (19) – (22) for three-point analysis). In the M step, an explicit equation is used to estimate the probability . The MLEs of  are further used to estimate *m*(*m *- 1)/2 recombination fractions between all possible marker pairs. In *m*-point analysis, parental diplotypes and gene orders can be incorporated in the model.

### Monte Carlo simulation

Simulation studies are performed to investigate the statistical properties of our model for simultaneously estimating linkage, parental diplotype and gene order in a full-sib family derived from two outbred parents. Suppose there are five markers of a known order on a chromosome. These five markers are segregating differently in order, 1:1:1:1, 1:2:1, 3:1, 1:1 and 1:1:1:1. The diplotypes of the two parents for the five markers are given in Table 1 and using these two parents a segregating full-sib family is generated. In order to examine the effects of parameter space on the estimation of linkage, parental diplotype and gene order, the full-sib family is simulated with different degrees of linkage (*r *= 0.05 vs. 0.20) and different sample sizes (*n *= 100 vs. 200).

As expected, the estimation precision of the recombination fraction depends on the marker type, the degree of linkage and sample size. More informative markers, more tightly linked markers and larger sample sizes display greater estimation precision of linkage than less informative markers, less tightly linked markers and smaller sample sizes (Tables 1 and 2). To save space, we do not give the results about the effects of sample size in the tables. Our model can provide an excellent estimation of parental linkage phases, i.e., parental diplotype, in two-point analysis. For example, the MLE of the probability (*p *or *q*) of parental diplotype is close to 1 or 0 (Table 1), suggesting that we can always accurately estimate parental diplotypes. But for two symmetrical markers (e.g., markers  and  in this example), two sets of MLEs,  = 1,  = 0 and  = 0,  = 1, give an identical likelihood ratio test statistic. Thus, two-point analysis cannot specify parental diplotypes for symmetrical markers even when the two parents have different diplotypes.

The estimation precision of linkage can be increased when a three-point analysis is performed (Table 2), but this depends on different marker types and different degrees of linkage. Advantage of three-point analysis over two-point analysis is more pronounced for partially than fully informative markers, and for less tightly than more tightly linked markers. For example, the sampling error of the MLE of the recombination fraction (assuming *r *= 0.20) between markers  and  from two-point analysis is 0.0848, whereas this value from a three-point analysis decreases to 0.0758 when combining fully informative marker  but increases to 0.0939 when combining partially informative marker . The three-point analysis can clearly determine the diplotypes of different parents as long as one of the three markers is asymmetrical. In our example, using either asymmetrical marker  or , the diplotypes of the two parents for two symmetrical markers ( and ) can be determined. Our model for three-point analysis can determine a most likely gene order. In the three-point analyses combining markers , markers  and marker , the MLEs of the probabilities of gene order are all almost equal to 1, suggesting that the estimated gene order is consistent with the order hypothesized.

To demonstrate how our linkage analysis model is more advantageous over the existing models for a full-sib family population, we carry out a simulation study for linked dominant markers. In two-point analysis, two different parental diplotype combinations are assumed: (1) [*aa*] [*oo*] × [*aa*] [*oo*] (*cis *× *cis*) and (2) [*ao*] [*oa*] × [*ao*] [*oa*] (*trans *× *trans*). The MLE of the linkage under combination (2), in which two dominant alleles are in a repulsion phase, is not as precise as that under combination (1), in which two dominant non-alleles are in a coupling phase [12]. For a given data set with unknown linkage phase, the traditional procedure for estimating the recombination fraction is to calculate the likelihood values under all possible linkage phase combinations (i.e., *cis *× *cis*, *cis *× *trans*, *trans *× *cis *and *trans *× *trans*). The combinations, *cis *× *cis *and *trans *× *trans*, have the same likelihood value, with the MLE of one combination being equal to the subtraction of the MLE of the second combination from 1. The same relationship is true for *cis *× *trans *and *trans *× *cis*. A most likely phase combination is chosen corresponding to the largest likelihood and a legitimate MLE of the recombination fraction (*r *≤ 0.5) [10].

For our data set simulated from [*aa*] [*oo*] × [*aa*] [*oo*], one can easily select *cis *× *cis *as the best estimation of phase combination because it corresponds to a larger likelihood and a smaller  (Table 3). Our model incorporating the parental diplotypes can provide comparable estimation precision of the linkage for the data from [*aa*] [*oo*] × [*aa*] [*oo*] and precisely determine the parental diplotypes (see the MLEs of *p *and *q*; Table 3). Our model has great advantage over the traditional model for the data derived from [*ao*] [*oa*] × [*ao*] [*oa*]. For this data set, the same likelihood was obtained under all possible four diplotype combinations (Table 3). In this case, one would select *cis *× *trans *or *trans *× *cis *because these two phase combinations are associated with a lower estimate of *r*. But this estimate of *r *(0.0393) is biased since it is far less than the value of 0.20 hypothesized. Our model gives the same estimation precision of the linkage for the data derived from [*ao*] [*oa*] × [*ao*] [*oa*] as obtained when the analysis is based on a correct diplotype combination (Table 3). Also, our model can precisely determine the parental diplotypes ( =  = 0 ).

**Table 3:** Comparison of the estimation of the linkage and parental diplotype between two dominant markers in a full-sib family of *n *= 100 from the traditional and our model

	Traditional model				Our model
		
	*cis *× *cis*	*cis *× *trans*	*trans *× *cis*	*trans *× *trans*	
Data simulated from *cis *× *cis*					
Correct diplotype combination	Correct	Incorrect	Incorrect	Incorrect	
Log-likelihood^*a*^	-46.2	-92.3	-92.3	-46.2	
under each diplotype combination	0.1981 ± 0.0446	0.5000 ± 0.0000	0.5000 ± 0.0000	0.8018 ± 0.0446	
Estimated diplotype combination	Selected				
under correct diplotype combination	0.1981 ± 0.0446				0.1982 ± 0.0446
Diplotype probability for parent P ()					1.0000 ± 0.0000
Diplotype probability for parent Q ()					1.0000 ± 0.0000
Data simulated from *trans *× *trans*					
Correct diplotype combination	Incorrect	Incorrect	Incorrect	Correct	
Log-likelihood^*a*^	-89.6	-89.6	-89.6	-89.6	
under each diplotype combination	0.8573 ± 0.1253	0.0393 ± 0.0419	0.0393 ± 0.0419	0.1426 ± 0.1253	
Estimated diplotype combination		Selected	Selected		
under correct diplotype combination				0.1426 ± 0.1253	0.1428 ± 0.1253
Diplotype probability for parent P ()					0.0000 ± 0.0000
Diplotype probability for parent Q ()					0.0000 ± 0.0000

^*a*^The log-likelihood values given here are those from one random simulation for each diplotype combination by the traditional model.

In three-point analysis, we examine the advantage of implementing linkage analysis with gene orders. Three dominant markers are assumed to have two different parental diplotypes combinations: (1) [*aaa*] [*ooo*] × [*aaa*] [*ooo*] and (2) [*aao*] [*ooa*] × [*aao*] [*ooa*]. The traditional approach is to calculate the likelihood values under three possible gene orders and choose one of a maximum likelihood to estimate the linkage. Under combination (1), a most likely gene order can be well determined and, therefore, the recombination fractions between the three markers well estimated, because the likelihood value of the correct order is always larger than those of incorrect orders (Table 4). However, under combination (2), the estimates of linkage are not always precise because with a frequency of 20% gene orders are incorrectly determined. The estimates of *r*'s will largely deviate from their actual values based on a wrong gene order (Table 4). Our model incorporating gene order can provide the better estimation of linkage than the traditional approach, especially between those markers with dominant alleles being in a repulsion phase. Furthermore, a most likely gene order can be determined from our model at the same time when the linkage is estimated.

**Table 4:** Comparison of the estimation of the linkage and gene order between three dominant markers in a full-sib family of *n *= 100 from the traditional and our model

MLE	Traditional model			Our model
		
				
Data stimulated from [*aaa*] [*ooo*] × [*aaa*] [*ooo*]				
Correct gene order	Correct	Incorrect	Incorrect	
Estimated best gene order (%^*a*^)	100	0	0	
	0.2047 ± 0.0422			0.2048 ± 0.0422
	0.1980 ± 0.0436			0.1985 ± 0.0434
	0.3245 ± 0.0619			0.3235 ± 0.0618
				0.9860 ± 0.0105
				0.0060 ± 0.0071
				0.0080 ± 0.0079
Data simulated from [*aao*] [*ooa*] × [*aao*] [*ooa*]				
Correct gene order	Correct	Incorrect	Incorrect	
Estimated best gene order (%^*a*^)	80	11	9	
	0.1991 ± 0.0456	0.8165 ± 0.1003	0.9284 ± 0.0724	0.2104 ± 0.0447
	0.1697 ± 0.0907	0.8220 ± 0.0338	0.1636 ± 0.0608	0.2073 ± 0.0754
	0.3218 ± 0.0755	0.2703 ± 0.0586	0.7821 ± 0.0459	0.2944 ± 0.0929
				0.9952 ± 0.0058
				0.0045 ± 0.0058
				0.0003 ± 0.0015

^*a*^The percents of a total of 200 simulations that have a largest likelihood for a given gene order estimated from the traditional approach. In this example used to examine the advantage of implementing gene orders, known linkage phases are assumed.

Our model is further used to perform joint analyses including more than three markers. When the number of markers increases, the number of parameters to be estimated will be exponentially increased. For four-point analysis, the speed of convergence was slow and the accuracy and precision of parameter estimation have been affected for a sample size of 200 (data not shown). According to our simulation experience, the improvement of more-than-three-point analysis can be made possible by increasing sample size or by using the estimates from two- or three-point analysis as initial values.

### A worked example

We use an example from published literature [18] to demonstrate our unifying model for simultaneous estimation of linkage, parental diplotype and gene order. A cross was made between two triple heterozygotes with genotype *AaVvXx *for markers ,  and . Because these three markers are dominant, the cross generates 8 distinguishable genotypes, with observations of 28 for *A*_-_/*V*_-_/*X*_-_, 4 for *A*_-_/*V*_-_/*xx*, 12 for *A*_-_/*vv*/*X*_-_, 3 for *A*_-_/*vv*/*xx*, 1 for *aa*/*V*_-_/*X*_-_, 8 for *aa*/*V*_-_/*xx*, 2 for *aa*/*vv*/*X*_- _and 2 for *aa*/*vv*/*xx*. We first use two-point analysis to estimate the recombination fractions and parental diplotypes between all possible pairs of the three markers. The recombination fraction between markers  and  is , whose the estimated parental diplotypes are [*Av*] [*aV*] × [*AV*] [*av*] or [*AV*] [*av*] × [*Av*] [*aV*]. The other two recombination fractions and the corresponding parental displotypes are estimated as , [*Vx*] [*vX*] × [*VX*] [*vx*] or [*VX*] [*vx*] × [*Vx*] [*vX*] and , [*AX*] [*ax*] × [*AX*] [*ax*], respectively. From the two-point analysis, one of the two parents have dominant alleles from markers  and  are repulsed with the dominant alleles from marker .

Our subsequent three-point analysis combines parental diplotypes and gene orders to estimate the linkage between these three dominant markers. The estimated gene order is . The MLEs of the recombination fractions are ,  and . The parental diplotype combination is [*XAV*] [*xav*] × [*XAv*] [*xaV*] or [*XAv*] [*xaV*] × [*XAV*] [*xav*]. The three-point analysis for these three markers by Ridout et al. [18] led to the estimates of the three recombination fractions all equal to 0.20. But their estimates may not be optimal because the effect of gene order on  was not considered.

## Discussion

Several statistical methods and software packages have been developed for linkage analysis and map construction in experimental crosses and well-structured pedigrees [2-6], but these methods need unambiguous linkage phases over a set of markers in a linkage group. For outcrossing species, such as forest trees, it is not possible to know exact linkage phases for any of two parents that are crossed to generate a full-sib family prior to linkage analysis. This uncertainty about linkage phases makes linkage mapping in outcrossing populations much more difficult than that in phase-known pedigrees [7,9].

In this article we present a unifying model for simultaneously estimating the linkage, parental diplotype and gene order in a full-sib family derived from two outbred parents. As demonstrated by simulation studies, our model is robust to different parameter space. Compared to the traditional approaches that calculate the likelihood values separately under all possible linkage phases or orders [9,10,18], our approach is more advantageous in three aspects. First, it provides a one-step analysis of estimating the linkage, parental diplotype and gene order, thus facilitating the implementation of a general method for analyzing any segregating type of markers for outcrossing populations in a package of computer program. For some short-generation-interval outcrossing species, we can obtain marker information from grandparents, parents and progeny. The model presented here allow for the use of marker genotypes of the grandparents to derive the diplotype of the parents. Second, our model for the first time incorporates gene ordering into a unified linkage analysis framework, whereas most earlier studies only emphasized on the characterization of linkage phases through a multilocus likelihood analysis [11,14,15]. Instead of a comparative analysis of different orders, we proposed to determine a most likely gene order by estimating the order probabilities.

Third, and most importantly, our unifying approach can significantly improve the estimation precision of the linkage for dominant markers whose alleles are in repulsion phase. Previous analyses have indicated that the estimate of the linkage between dominant markers in a repulsion phase is biased and imprecise, especially when the linkage is not strong and when sample size is small [12]. There are two reasons for this: (1) the linkage phase cannot be correctly determined, and/or (2) there is a fairly high possibility (20%) of detecting a wrong gene order. Our approach provides more precise estimates of the recombination fraction because correct parental diplotypes and a correct gene order can be determined.

Our approach will be broadly useful in genetic mapping of outcrossing species. In practice, a two-point analysis can first be performed to obtain the pairwise estimates of the recombination fractions and using this pairwise information markers are grouped based on the criteria of a maximum recombination fraction and minimum likelihood ratio test statistic [2]. The parental diplotypes of markers in individual groups are constructed using a three-point analysis. With a limited sample size available in practice, we do not recommend more-than-three-point analysis because this would bring too many more unknown parameters to be precisely estimated. If such an analysis is desirable, however, one may use the results from these lower-point analyses as initial values to improve the convergence rate and possibly the precision of parameter estimation.

In any case, our two- and three-point analysis has built a key stepping stone for map construction through two approaches. One is the least-squares method, as originally developed by Stam [5], that can integrate the pairwise recombination fractions into reconstruction of multilocus linkage map. The second is to use the hidden Markov chain (HMC) model, first proposed by Lander and Green [2], to construct genetic linkage maps by treating map construction as a combinatorial optimization problem. The simulated annealing algorithm [19] for searching for optima of the multilocus likelihood function need to be implemented for the HMC model. A user-friendly package of software that is being written by the senior author will implement two- and three-point analyses as well as the algorithm for map construction based on the estimates of pairwise recombination fractions. This software will be online available to the public.

Our maximum likelihood-based approach is implemented with the EM algorithm. We also incorporate the Gibbs sampler [20] into the estimation procedure of the mixture model for the linkage characterizing different parental diplotypes and gene orders of different markers. The results from the Gibbs sampler are broadly consistent with those from the EM algorithm, but the Gibbs sampler is computationally more efficient for a complicated problem than the EM algorithm. Therefore, the Gibbs sampler may be particularly useful when our model is extended to consider multiple full-sib families in which the parents may be selected from a natural population. For such a multi-family design, some population genetic parameters describing the genetic structure of the original population, such as allele frequencies and linkage disequilibrium, should be incorporated and estimated in the model for linkage analysis. It can be anticipated that the Gibbs sampler will play an important role in estimating these parameters simultaneously along with the linkage, linkage phases, and gene order.

## Authors' contributions

QL derived the genetic and statistical models and wrote computer programs. YHC participated in the derivations of models and statistical analyses. RLW conceived of ideas and algorithms, and wrote the draft. All authors read and approved the final manuscript.
